# Development, dissemination, and applications of a new terminological resource, the Q-Code taxonomy for professional aspects of general practice/family medicine

**DOI:** 10.1080/13814788.2017.1404986

**Published:** 2017-12-15

**Authors:** Marc Jamoulle, Melissa Resnick, Julien Grosjean, Ashwin Ittoo, Elena Cardillo, Robert Vander Stichele, Stefan Darmoni, Marc Vanmeerbeek

**Affiliations:** ^a^ Department of General Practice, University of Liège Liège Belgium; ^b^ Health Sciences Center, University of Texas Houston TX USA; ^c^ Department of Information and Medical Informatics (D2IM), University of Rouen Rouen France; ^d^ HEC School of Management, University of Liège Liège Belgium; ^e^ Italian Institute of Informatics and Telematics, National Research Council Cosenza Italy; ^f^ Heymans Institute, University of Ghent Ghent Belgium

**Keywords:** General practice, primary healthcare, terminology as topic, semantic web, qualitative research, classification

## Abstract

**Background:** While documentation of clinical aspects of General Practice/Family Medicine (GP/FM) is assured by the International Classification of Primary Care (ICPC), there is no taxonomy for the professional aspects (context and management) of GP/FM.

**Objectives:** To present the development, dissemination, applications, and resulting face validity of the Q-Codes taxonomy specifically designed to describe contextual features of GP/FM, proposed as an extension to the ICPC.

**Development:** The Q-Codes taxonomy was developed from Lamberts’ seminal idea for indexing contextual content (1987) by a multi-disciplinary team of knowledge engineers, linguists and general practitioners, through a qualitative and iterative analysis of 1702 abstracts from six GP/FM conferences using Atlas.ti software. A total of 182 concepts, called Q-Codes, representing professional aspects of GP/FM were identified and organized in a taxonomy.

**Dissemination:** The taxonomy is published as an online terminological resource, using semantic web techniques and web ontology language (OWL) (http://www.hetop.eu/Q). Each Q-Code is identified with a unique resource identifier (URI), and provided with preferred terms, and scope notes in ten languages (Portuguese, Spanish, English, French, Dutch, Korean, Vietnamese, Turkish, Georgian, German) and search filters for MEDLINE and web searches.

**Applications:** This taxonomy has already been used to support queries in bibliographic databases (e.g., MEDLINE), to facilitate indexing of grey literature in GP/FM as congress abstracts, master theses, websites and as an educational tool in vocational teaching,

**Conclusions:** The rapidly growing list of practical applications provides face-validity for the usefulness of this freely available new terminological resource.

KEY MESSAGES

- A taxonomy for professional aspects of GP/FM is proposed as an extension to the ICPC classification.
- 182 Q-Codes are available as an online semantic web resource in 10 languages.
- Face validity of this new taxonomy is illustrated by the rapidly growing list of current applications.

## Introduction

Several attempts have been made to define the distinct concepts of general practice and family medicine (GP/FM), as in statements of professional organizations [1–5] and medical textbooks [6–11]. These definitions have been reviewed previously [12] in an attempt to delineate the scope of the field.

For the documentation of clinical data, the International Classification of Primary Care (ICPC-2) is used [13], often in conjunction with the International Classification of Diseases (ICD).

ICPC-2 has been developed to identify reasons for encounters, such as symptoms, acts performed or requested and diagnosis in daily practice. However, it was not constructed to reflect the professional context of GP/FM, such as organizational, managerial, ethical, environmental, educational, and investigational factors.

The absence of adequate normalized keywords for identifying specific professional contextual features of GP/FM is an impediment to proper indexing of grey literature in GP/FM.

More than 50% of the scientific outputs of general practitioners at congresses are never published [14,15]. Since McWhinney's famous initiative FAMLI [16,17], there is no knowledge management system dedicated to GP/FM grey literature (research which has not been published in academic journals, nor indexed by the major bibliographic databases) [18]. Consequently, study reports, congress abstracts, or collections of dissertations are often not globally available to researchers and practitioners, due to this lack of indexation [19]. For example, the Wonca Europe website maintains a database of more than 30 000 abstracts of conferences in GP/FM. However, these contents are not indexed, which hinders their utility to foster research and support the daily operations of GP/FM.

A taxonomy of professional contextual features of GP/FM has been developed to address this issue using present day technology for information storage and retrieval [20], such as machine learning, semantic web technologies and natural language processing (NLP).

This new taxonomy was named Q-Codes, in honour of the late Henk Lamberts, a Dutch professor of family medicine (University of Amsterdam), who proposed to use the letter Q (not yet used in ICPC) for the development of a rudimentary coding system for his departmental research library.

## Aim

The aim of this background paper is: (i) to shortly describe the development of the Q-Codes taxonomy, designed to cover the professional contextual aspects of GP/FM; (ii) to present its dissemination tools available for accessing the taxonomy; and (iii) to describe a number of recent applications, as an exploration of the face-validity of this new terminological resource for primary care.

## Development of the taxonomy

A qualitative and iterative analysis of 1702 abstracts in English or French from six GP/FM congresses held between 2007 and 2014 (Table 1) has been performed from a practicing GPs perspective, in a bottom-up approach, to identify relevant concepts focusing on the professional features of GP/FM (e.g. teaching, ethics, or environmental hazard issues). The choice of conferences was pragmatic. Abstracts needed to be available for analysis by the first author (MJ) in due time before the conference, so that the results could be presented and discussed with attendees [21]. The study of Kruschinski et al., on the content of 614 abstracts of the European General Practice Research Network used a similar methodology to delineate the aspects of GP/FM and was reused in the development process of the taxonomy [22].

**Table 1.** Sources and languages of the abstracts analysed.

Year	Data origin	Language(s)	Source	*n*
2007	Online. Access to reviewer pages before the congress	English	Wonca Europe conference	998
2013	Print, received during the congress	Portuguese	Portuguese 18th National Conference of Family Medicine	128
2013	Excel file, abstracts received after the congress	French	Congres de la Confédération des Généralistes Enseignants (CNGE)	205
2014	Online. Access to reviewer pages before the congress	French	Congres de la Confédération des Généralistes Enseignants (CNGE)	289
2014	Print, published in Primary care, 2014	English, French, German	SwissFamilyDocs 2014	45
2014	Excel file	French	Belgian Congress 2014	37
2010	Print, published Family Practice 27(4) 459:67	English	EGPRN Study	614

Starting point for the analysis of the congress abstracts were the original Q-Codes created by H. Lamberts. To identify what is really at stake and respect the bottom-up approach, only themes addressed by GPs during the conferences have been classified. The data was analysed in a grounded theory approach with Atlas.ti software (http://atlasti.com/). Each abstract is read and code attributed along the classification ICPC-2 for clinical aspects and with a Q-Code for emerging professional contextual aspects. By grouping these identified concepts and their Q-Codes into subcategories and categories, gradually the taxonomy (a hierarchical ontological structure) takes shape. Cimino’s standard set of desiderata for terminological resources was applied [23]. For a full description of this development, the reader is referred to a prior publication [24].

Each concept was illustrated by a carefully chosen sample of pertinent articles, to illustrate and deepen the understanding of the each individual Q-Code. Each Q-Code was linked to other terminological systems reference terminologies, such as BabelNet.org, DBpedia, and to carefully chosen controlled vocabulary MeSH and to a selected bibliography in open access.

Twenty-four GPs and terminology experts from 10 countries were involved in the translation of the terms and scope notes into ten languages, thereby contributing to the further validation of the concept description of each Q-Code.

In the end, a taxonomy of 182 Q-Codes was created to represent professional contextual features of GP/FM. There are eight domains in the taxonomy: doctor’s issues, patient’s issues, patient categories, structure, knowledge management, and research and development. The two remaining domains are planetary health issues and medical ethics. Each domain is further divided into categories and subcategories (see the electronic annex for a full list of the 182 codes).

## Dissemination of the taxonomy

The taxonomy with 182 Q-Codes in 10 languages was published as a semantic web resource on the multilingual, multi-terminology portal HETOP after free inscription (http://www.hetop.eu) [25] and in uniform resource identifier (URI) format (Table 2).

**Table 2.** Free access to ICPC-2 and Q-Codes in URI format. To change the language; change the ISO 639 code for the language.

• To reach the hierarchy
ICPC-2: http://www.hetop.org/hetop/?la=en&rr=CIP_C_ARBO&tab =1
ICPC-2 process: http://www.hetop.org/hetop/?la=en&rr=CIP_C_ARBOPROC&tab =1
Q-Codes: http://www.hetop.eu/hetop/Q?la=en&rr=CGP_CO_Q&tab =1
• To reach each rubrics
ICPC: http://www.hetop.org/hetop/?la=en&rr=CIP_D_A01
ICPC process: http://www.hetop.org/hetop/?la=en&rr=CIP_P_30
Q-Codes http://www.hetop.eu/hetop/Q?la=en&rr=CGP_QC_QC1

Language ISO 639 codes between brackets; Portuguese (pt), Spanish (es), English (en), French (fr), Dutch (nl), Korean (ko), Vitenamese (vi), Turkish (tr), Georgian (ka), German (de).

## Applications of the taxonomy

Numerous practical are currently in use or under development (see http://3cgp.docpatient.net for a constantly updated overview). We will limit ourselves here to describe examples of recent applications for three domains: facilitating bibliographic searching; facilitating indexing of congress abstracts and dissertations; and e-learning applications.

## Facilitating bibliographic searching

Within the HeTOP website, a search and retrieval engine was built, which allows for multilingual bibliographic queries on PubMed. These queries facilitate the search for relevant literature on issues in primary care.

**Figure 1.**
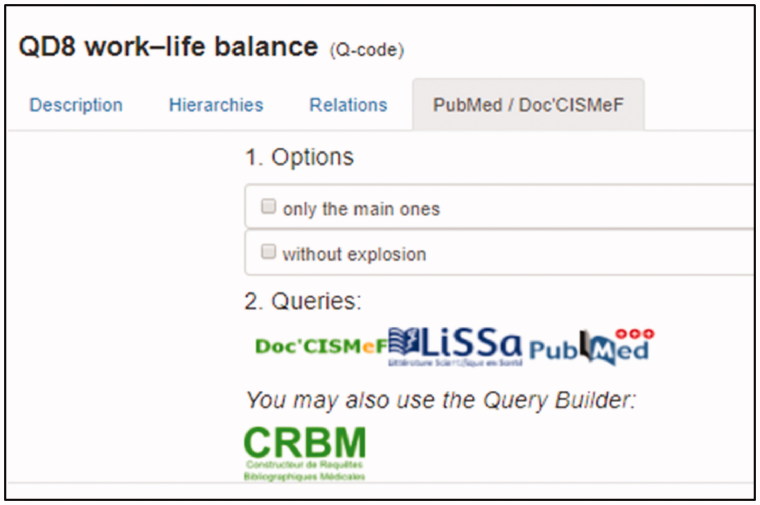
The multilingual Inforoute info-button of HeTOP allows access to an automatic query on CISFMef and LiSSa (French knowledge bases) and on PubMed.

We will illustrate this with an example. Suppose that a medical student or general practitioner is interested in studies pertaining to the concept of ‘work–life balance.’ This concept is a Q-Code, but not a keyword in medical subject headings (MeSH), which makes it difficult to conduct a search in MEDLINE. By clicking on the Q-Code QD8 on http://www.hetop.eu/Q, relevant MeSH keywords will be suggested, with detailed information on their scope, and relationships in the MeSH hierarchy. In addition, a relevant search profile for this concept will be automatically provided, turning 182 Q-Codes into search filters for LiSSa (http://www.lissa.fr), a French publications database and MEDLINE (PubMed; Figure 1).

Trainees in GP/FM have used this search engine to explore concepts at stake during a consultation, and to have a prepared bibliography search for each important theme used in daily practice. An example of this approach is given in http://docpatient.net/3CGP/QC/clinical_exercise.htm

## Indexation of dissertations and congress abstracts

Q-Codes taxonomy and ICPC-2 are used to index the master theses in GP/FM in French-speaking Belgium (see http://ww.mgtfe.be (in French) and at the Department of General Practice of the University of Coimbra, Portugal). The results of long working hours devoted to the realization of an exciting final master thesis will remain unread, if not properly indexed. Students are offered ICPC, the Q-Code taxonomy, an indexing tool from the HeTOP portal, and a user guide to assist them in the task to index their work [26].

This approach is now reused by researchers from Houston University, Texas, US, for indexing of question-answer pairs in telehealth Brazil and by the Brazilian Society of Family and Community Medicine (SBMFC) to support the indexing of the congress abstracts of the next SBMFC congress (http://www.cbmfc2017.com.br/trabalhos/) [27].

Indexing of congress abstracts allows congress participants to identify more quickly particular contributions of interest during the conference. Researchers not attending the conference can also more easily pinpoint interesting contributions and their authors.

In addition, indexing all abstracts allows for a careful study of the scope and main points of the conference. As an example, we present shortly the results of such an analysis of the 212 abstracts of the congress of the French Society of teachers in GP/FM (CNGE), held in Lille in 2014. ICPC-2 was used to index clinical issues, and the Q-Codes taxonomy for professional contextual features. The distribution of themes of the QT domain (training and teaching) gives an insight in the abstracts presented (Figure 2). In the abstracts presented at this congress, 45 address the general issue of quality assurance, 14 are about EBM and 38 about guidelines, etc.

**Figure 2.**
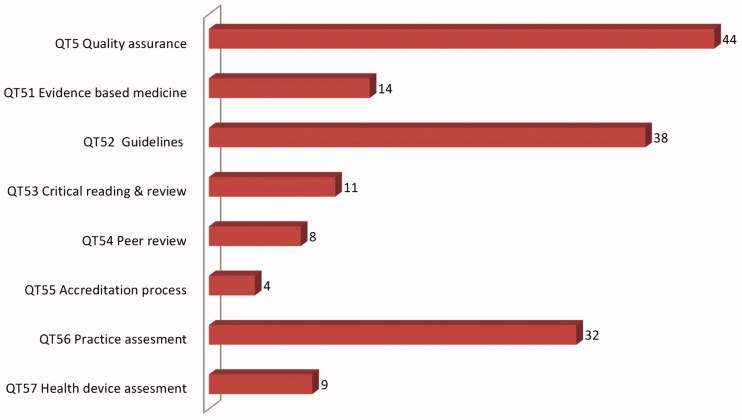
Themes addressing the issue of quality in the domain QT knowledge translation encountered in the 211 abstracts of the CNGE congress in 2014, Lille, France.

In France, a multi-terminology concepts extractor (ECMT) is created for the full automation of the indexing process for conference abstracts, dissertations, and abstracts from the French LiSSa base [28]. A project to combine the Q-Codes taxonomy with this tool for application in the domain of GP/FM is initiated.

## E-learning applications

In Vietnam, the Q-Codes taxonomy has been used in an e-learning programme for GP/FM students (http://3cgp.docpatient.net/action-research/q-codes-in-an-e-learning-program-in-vietnam). In this project, natural language processing techniques are used to guide the students to the right Q-Codes for their documentation needs.

### Potential impacts for practice

#### A taxonomy inspired by MeSH, focused on GP/FM

The Q-Codes taxonomy for professional aspects of GP/FM has been developed as a new terminological resource for primary care. It is proposed as an extension to the clinically oriented International Classification for Primary Care (ICPC). This taxonomy is available on the web on http://www.hetop.eu/Q (free access) and in URI format (Table 1), as a professional terminological resource in 10 languages, and fit for semantic web applications.

This taxonomy and its tools of dissemination are available at the point of care, to practicing physicians, researchers, trainers and trainees in GP/FM, as an illustration of the extent and complexity of this profession.

A growing list of recent applications of this taxonomy illustrates its rapid uptake, and provides face-validity to its usefulness for scientific communications in the field of GP/FM, by facilitating indexing of dissertations, master theses and congress abstracts, and facilitating bibliographic searches in grey literature repositories of primary care and in general bibliographic systems such as MEDLINE.

The Q-Codes taxonomy has been developed from a strong empirical basis in a bottom-up approach, and applying terminological expertise and sound qualitative methods. To provide free access to the taxonomy, a database was published on the web, created with a standardized terminological structure and advanced semantic web techniques. Great effort was invested in linking and mapping the newly identified Q-Codes to existing concepts in existing terminologies, both linguistic (Babelnet) and conceptual (PubMed).

Further ontological research is needed to determine whether the two main rules of taxonomical thinking have been respected: completeness (all identified) and exclusivity (one place for each concept) [29]. A taxonomy is never complete and must evolve with the discipline. In this development process, we stopped at 182 Q-Codes, but future congress will certainly reveal new concepts.

The value of a taxonomy is determined by the quality and speed of the maintenance and updating process. Although Q-Codes have not been officially endorsed by the World Organization of Family Doctors International Classification Committee (WICC), the maintenance will be handled by a working group of WICC (http://www.ph3c.org/Q) who are also the custodians of ICPC-2.

#### An attempt to design a draft of the table of content of GP/FM

To the best of our knowledge, this is the first systematic, internationally accepted description of professional aspects in GP/FM. A historic predecessor was FAMLI, a short-lived extension of MeSH, available only in print and floppy disks, operational since 1980, and governed by Ian McWhinney (a founding father of GP/FM) [16,17]. This effort was abandoned in 1992 [30]. This approach to the doctor’s professional context should not be confused with the contextual approach of the patient’s universe, as developed by Schrans [31], who studied the elements of the patient’s life context (ideas, concerns, and expectations) that influence his or her state of health, the health problems offered to the doctor, with substantial impact on the process of care.

One could argue that the development of a profession-specific taxonomy for professional aspects of GP/FM is unnecessary, as the aims for such a taxonomy could be met with existing resources, such medical subject headings (MeSH). However, the experience gained during the development of the taxonomy has shown that many profession-specific concepts are not properly represented in MeSH [32]. Furthermore, the learning curve for the correct use of a general and vast thesaurus is steeper than for the use of a simple profession-specific taxonomy.

#### A tool supporting the future of multicultural GP/FM

The feasibility to translate the Q-Codes labelling and scope notes in ten languages illustrates that the taxonomy is robust to cultural diversity. The rapid acceptance of this new terminological tool and its integration in some recent applications is also an indication of face-validity. However, more in-depth research is needed to underpin the validity of this taxonomy for its intended uses. Studying the impact of the use of the taxonomy in search and indexing efficiency of medical students, general practitioners, librarians, and automated systems alike will be the next steps.

The use of automatic ontology learning techniques, relying on term extraction and semantic relation extraction, should be explored to identify concepts and relations from literature and incorporate them in the taxonomy, and facilitate the maintenance process.
